# Highly Conductive and Water-Swelling Resistant Anion Exchange Membrane for Alkaline Fuel Cells

**DOI:** 10.3390/ijms20143470

**Published:** 2019-07-15

**Authors:** Qianqian Ge, Xiang Zhu, Zhengjin Yang

**Affiliations:** 1 Polymer Composites Group, School of Chemistry & Chemical Engineering, Anhui University, Hefei 230601, China; 2 CAS Key Laboratory of Soft Matter Chemistry, Collaborative Innovation Center of Chemistry for Energy Materials, School of Chemistry and Materials Science, University of Science and Technology of China, Hefei 230026, China

**Keywords:** hyperbranched polymer, crosslinking, alkaline fuel cells, ionic conductivity, water swelling

## Abstract

To ameliorate the trade-off effect between ionic conductivity and water swelling of anion exchange membranes (AEMs), a crosslinked, hyperbranched membrane (C-HBM) combining the advantages of densely functionalization architecture and crosslinking structure was fabricated by the quaternization of the hyperbranched poly(4-vinylbenzyl chloride) (HB-PVBC) with a multiamine oligomer poly(*N*,*N*-Dimethylbenzylamine). The membrane displayed well-developed microphase separation morphology, as confirmed by small angle X-ray scattering (SAXS) and transmission electron microscopy (TEM). Moreover, the corresponding high ionic conductivity, strongly depressed water swelling, high thermal stability, and acceptable alkaline stability were achieved. Of special note is the much higher ratio of hydroxide conductivity to water swelling (33.0) than that of most published side-chain type, block, and densely functionalized AEMs, implying its higher potential for application in fuel cells.

## 1. Introduction

Polymer electrolyte membrane fuel cells recently appeared as one of the most promising energy-conversion devices owing to their simplified operation, higher power density, and easier maintenance over conventional fuel cells with liquid solution as electrolyte [1,2]. The commonly used solid polymer electrolyte is a perfluorinated sulfonic acid-based membrane known as Nafion, which shows high proton conductivity, excellent mechanical properties, and good chemical stability [3,4]. Despite the extraordinary performance of proton exchange membrane fuel cells (PEMFCs) assembled with Nafion, the strong acidic conditions restrict the utilization of highly stable catalysts, for example, platinum or platinum-containing metal alloys [5,6]. To improve these deficiencies while offering a nice alternative to acidic systems, alkaline fuel cells (AFCs) operating at high pH, permit the usage of non-platinum catalysts [5,7,8] such as silver, cobalt, or nickel and, therefore, has received considerable attention over the past few decades. Additionally, AFCs also exhibit distinct advantages over PEMFCs in terms of faster oxygen reduction kinetics and lower crossover of fuels owing to the opposite direction of electroosmotic drag [5,9,10]. However, exploring the alkaline electrolyte, that is, anion exchange membranes (AEMs), which is the key component acting as a separator between oxidant and fuel chambers and a conductor of hydroxide ions [11,12,13], with high conductivity, lower swelling, improved mechanical, and chemical stability compared with Nafion, is an important technical challenge.

High conductivity, which reflects the transporting efficiency of anions, is considered as the fundamental performance indicator for AEMs. However, the intrinsic lower mobility of OH^−^ and the less-developed microphase-separated morphology of aromatic polymers compared with perfluorinated structures naturally lead to lower ionic conductivity in AEMs compared with well-known proton exchange membranes (PEMs). To explore alternative AEMs with high ionic conductivity, a significant amount of research [14,15] has been carried out. Among which, increasing ion exchange capacity (IEC) values of ionomers is the most common and convenient strategy. However, high ionic conductivity obtained in this way is always at the expense of severe water uptake and a concomitant decline in the mechanical strength, especially at elevated temperatures [16,17]. Consequently, IEC values are typically restricted to be at a moderate level. Another reasonable route is incorporating functional groups with stronger basicity such as quaternary guanidinium groups [18] and quaternary 1,8-Diazabicyclo[5.4.0]undec-7-ene [19], aiming to enhance the dissociation ability and the simultaneous increase in ionic conductivity. However, limited success in increasing conductivity was achieved in this way. 

Notably, the subsequent investigation indicates that regulating the configuration of polymer backbones and the arrangement of ionic functional groups are effective strategies for improving ionic conductivity [5,20,21]. With respect to the configuration of polymer backbones, block copolymers are found to have well-defined hydrophilic–hydrophobic phase separated morphology [3,17,22,23,24]. Additionally, it is verified that positioning the functional groups on side chains rather than backbones can largely enhance their mobility to aggregate into ionic clusters and promote the microphase separation [9,21,25,26]. Moreover, decreasing the distance between the functional groups, that is, densely functionalization based on the block or side-chain type architecture, can further promote microphase separation and enhance the ionic conductivity [1,16,23,27,28,29]. However, most traditional densely functionalization strategies involved preparing multi-cation precursors via multi-steps, complicated synthesis, and purification procedures, which adds to the production cost and complexity. Our previous work reported the synthesis of hyperbranched oligomer poly-4-vinylbenzyl chloride (HB-PVBC) via one-step atom transfer radical polymerization (ATRP) reaction [30], which was a defining moment in hyperbranched AEM synthesis. The quaternization of HB-PVBC is expected to yield AEMs with ionic functional groups densely arranged. This specific structure is anticipated to provide a readily and effective strategy for preparing densely functionalized AEM with clear microphase separated morphology and high conductivity.

Though great progress has been acquired for increasing ionic conductivity, maintaining depressed water swelling at the same time remains challenging, especially at elevated temperatures [7]. To get over this dilemma between ionic conductivity and water swelling, significant research attention has been rendered and it has been approved that crosslinking is an effective strategy to obtain mechanically robust AEMs [2,4,29,30,31,32,33]. For instance, the crosslinking membrane CBQAPPO-3 exhibits the highest hydroxide conductivity of 33 mS cm^−1^ at room temperature and, simultaneously, benefitting from the crosslinking structure, much lower water uptake of 47.0% and linear swelling ratio (LSR) of 12.3% in comparison with that of the uncrosslinked counterpart BQAPPO-0.23 (water uptake = 120.4% and LSR = 24.1%) were acquired, leading to improved mechanical strength from 6.55 MPa to around 23 MPa [29,34]. More recently, cross-linking was also employed to toughen AEMs. As expected, the crosslinked membrane exhibits the significant improvement in water uptake (less than 7%), LSR (8%–5%, much smaller than uncrosslinked membranes) and tensile strength (higher than 18 MPa) [33]. The results showed that the crosslinking structure provides an efficient and convenient route for preparing membranes with much lower water swelling and the corresponding improved mechanical property. 

Thus, we wonder whether the combination of high cation density and crosslinking could lead to AEMs with high ionic conductivity and depressed water swelling. To verify this hypothesis, we designed and fabricated crosslinked, hyperbranched AEMs (defined as C-HBM) by crosslinking the hyperbranched oligomer HB-PVBC with a multiamine poly (*N*,*N*-Dimethylbenzylamine), aiming to combine the advantages of the densely functionalization architecture and crosslinking. The properties of the membrane such as microphase separated morphology, water uptake, LSR, ionic conductivity, alkaline stability, and thermal properties were thoroughly investigated.

## 2. Results and Discussion

### 2.1. Synthesis and Characterization

**Scheme 1:**
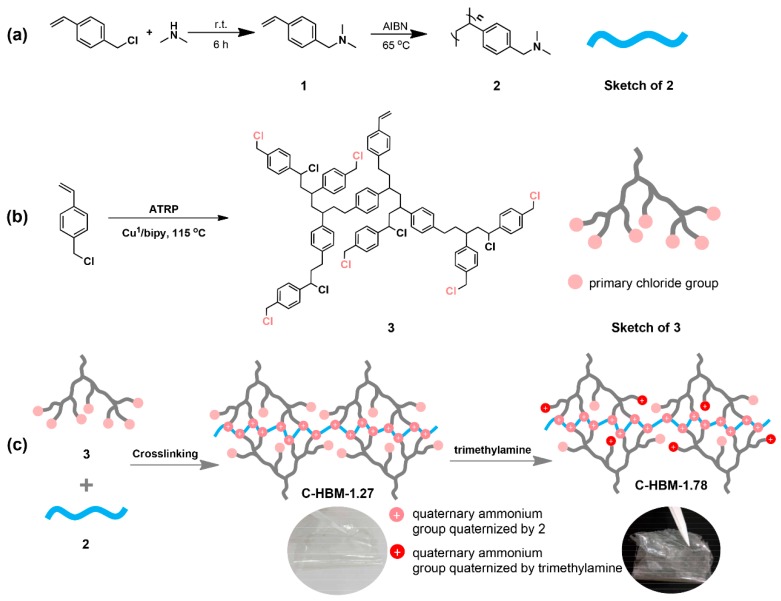
Schematic synthesis of the quaternization reagent poly(*N*,*N*-Dimethylbenzylamine), the hyperbranched oligomer poly-4-vinylbenzyl chloride (HB-PVBC) and the crosslinked, hyperbranched membrane C-HBM-1.78 (ion exchange capacity (IEC)=1.78 g^−1^). (**a**) Synthesis of the monomer 1 (*N*,*N*-Dimethylbenzylamine) through the amination of 4-vinylbenzyl chloride with dimethyl amine, and polyamine 2 (poly(*N*,*N*-Dimethylbenzylamine )) via radical polymerization; (**b**) synthesis of the hyperbranched oligomer HB-PVBC via atom transfer radical polymerization (ATRP) technique; and (**c**) the quaternization of HB-PVBC with poly(*N*,*N*-Dimethylbenzylamine) to yield densely functionalized ionomer C-HBM-1.27; further quaternization of C-HBM-1.27 with trimethylamine leads to membrane C-HBM-1.78 with a higher IEC value. AIBN, 2,2’-azodiisobutyronitrile.

**Figure 1:**
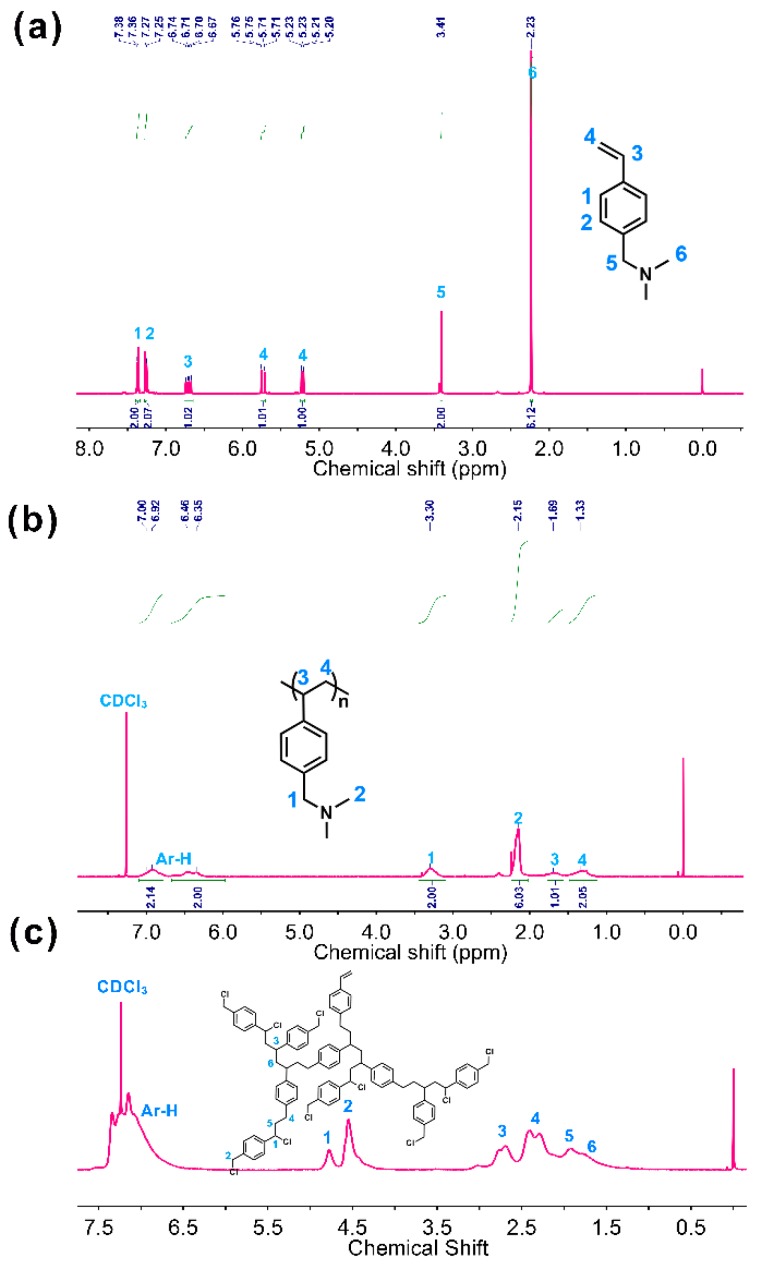
Proton nuclear magnetic resonance (^1^H NMR) spectra of (**a**) the monomer *N*,*N*-Dimethylbenzylamine, (**b**) polyamine poly(*N*,*N*-Dimethylbenzylamine), and (**c**) the hyperbranched oligomer HB-PVBC.

For the synthesis of *N*,*N*-Dimethylbenzylamine (Scheme 1a), 4-vinylbenzyl chloride was first dropped into an excessive amount of dimethyl amine aqueous solution to alleviate the formation of quaternary ammonium salt by-product. The mixture is then extracted and vacuum distilled to produce purified *N*,*N*-Dimethylbenzylamine. The purity and chemical structure is identified by proton nuclear magnetic resonance (^1^H NMR) analysis, as shown in Figure 1a. The expected chemical shifts and intensities for *N*,*N*-Dimethylbenzylamine were observed accordingly. The appearance of the peak at 2.2 ppm arising from methyl groups along with the shift of benzyl methylene groups from 4.3 ppm to 3.4 ppm indicates that the benzylic chloride groups has been successfully aminated. ^1^H NMR (400 MHz, CDCl_3_) δ 7.37 (d, *J* = 8.1 Hz, 2H), 7.26 (d, *J* = 8.1 Hz, 2H), 6.71 (dd, *J* = 17.6, 10.9 Hz, 1H), 5.73 (dd, *J* = 17.6, 0.9 Hz, 1H), 5.22 (dd, *J* = 10.9, 0.9 Hz, 1H), 3.41 (s, 2H), 2.23 (s, 6H).

**Figure 2:**
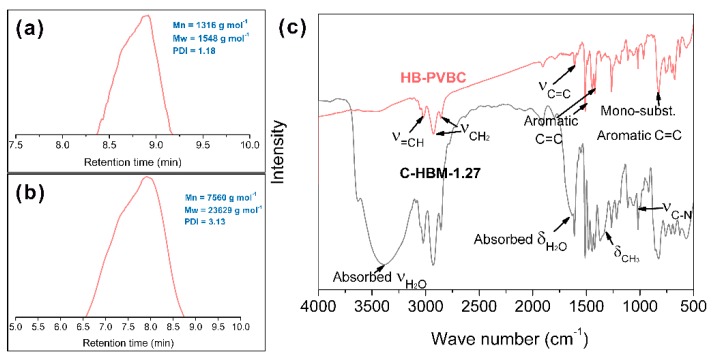
Gel permeation chromatography (GPC) traces of polyamine poly(*N*,*N*-Dimethylbenzylamine) (**a**) and the hyperbranched oligomer HB-PVBC (**b**); IR spectra of oligomer HB-PVBC and membrane C-HBM-1.27 (**c**). PDI, polydispersity.

The purified *N*,*N*-Dimethylbenzylamine was subjected to radical polymerization initiated by 2,2’-azodiisobutyronitrile (AIBN) to produce the polyamine poly(*N*,*N*-Dimethylbenzylamine) (Scheme 1a). Gel permeation chromatography (GPC) and ^1^H NMR techniques were used to monitor the structure. As shown in Figure 1b, the disappearance of vinyl groups at 5.2 ppm, 5.7 ppm, and 6.7 ppm within *N*,*N*-Dimethylbenzylamine in combination with the appearance of new peaks at 1.0−2.5 ppm assigned to methylene and methylidyne protons demonstrate the successful polymerization of *N*,*N*-Dimethylbenzylamine. ^1^H NMR (400 MHz, CDCl_3_) δ 6.96 (d, *J* = 30.0 Hz, 2H), 6.40 (d, *J* = 45.8 Hz, 2H), 3.30 (s, 2H), 2.15 (s, 6H), 1.69 (s, 1H), 1.33 (s, 2H). The molecular weight and polydispersity of poly(*N*,*N*-Dimethylbenzylamine) determined by GPC are 1316 g mol^−1^ and 1.18, respectively (Figure 2a). 

The hyperbranched HB-PVBC was synthesized according to our previous procedure [30] (Scheme 1b) and its chemical structure was verified by ^1^H NMR (Figure 1c). As estimated by GPC shown in Figure 2b, the molecular weight of HB-PVBC is 7560 g mol^−1^. As shown in Scheme 1b, all the primary chloride groups are densely distributed surrounding the oligomer HB-PVBC. The quaternization of primary chloride groups with tertiary amine is expected to endow the resulting ionomer with densely distributed functional groups. Herein, the polyamine poly(*N*,*N*-Dimethylbenzylamine) is utilized as the quaternization agent to yield densely functionalized AEM, thereby promoting the aggregation of ionic domains to form interconnected hydrophilic channels. Figure 2c shows the reflectance Fourier transform infrared (FTIR) spectra of the oligomer HB-PVBC and membrane C-HBM-1.27 (the titrated IEC value of 1.27 mmol g^−1^). The peak at around 3400 cm^−1^ arising from the stretching vibration of absorbed water molecules of quaternary ammonium salt groups is detected, confirming the successful implementation of quaternization and crosslinking. The titrated IEC value of 1.27 mmol g^−1^ further confirmed the successful functionalization of HB-PVBC. The membrane C-HBM-1.27 at fully hydrated state displayed tensile strength (TS) of 13.37 MPa and elongation at break (Eb) of 3.31% tested on dynamic mechanical analyzer. Immersing the membrane in trimethylamine aqueous solution at room temperature for 24 h can further quaternize the residual primary chloride groups, leading to an increased IEC of 1.78 mmol g^−1^. The yield membrane shows similar mechanical properties (TS = 12.37 MPa, Eb = 5.20%), and is denoted as C-HBM-1.78. Obviously, the mechanical property is inferior to most published cross-linking membranes [4,31,34], which is probably ascribed to the rigid structure of HB-PVBC and the multiaimne poly(*N*,*N*-Dimethylbenzylamine). As higher ionic content, that is, IEC value, generally leads to higher ionic conductivity, membrane C-HBM-1.78 is chosen for thorough investigation in the following section.

### 2.2. Membrane Morphology

**Figure 3:**
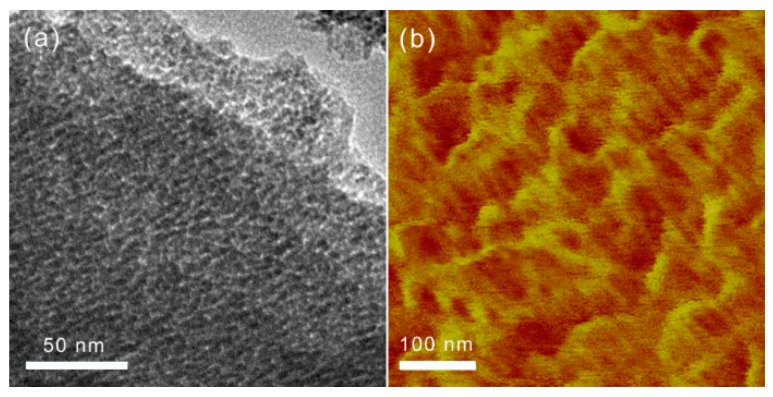
Microphase separated morphology images regarding (**a**) atomic force microscopy (AFM) and (**b**) transmission electron microscopy (TEM) of membrane C-HBM-1.78.

Transmission electron microscopy (TEM) was carried out for membrane C-HBM-1.78 stained with iodine ions. As shown in Figure 3a, the dark areas represent the soft hydrophilic regions mainly composed of water and quaternary ammonium clusters, while the brighter ones correspond to hard hydrophobic matrix mainly composed of aromatic rings [8]. Well-developed microphase separation morphology with interconnected ionic channels is observed for C-HBM-1.78 throughout the view. Further observation regarding atomic force microscopy (AFM, Figure 3b) confirms the formation of well-defined microphase separated morphology.

**Figure 4:**
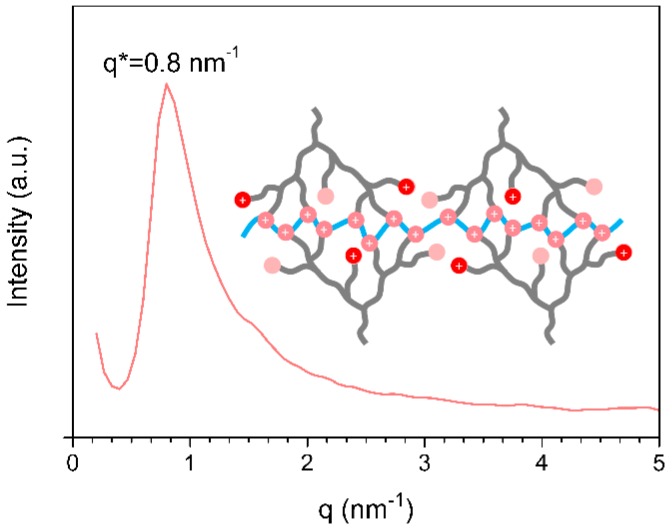
Small-angle X-ray scattering (SAXS) profile for membrane C-HBM-1.78 measured at room temperature under dry state (the insert is the schematic chemical structure of C-HBM-1.78).

Quantitative information on the size of the ionic clusters was obtained from the small-angle X-ray scattering (SAXS) results (Figure 4). A distinct peak at 0.8 nm^−1^ (corresponding to an interdomain Bragg spacing of 7.9 nm) [23] emerged in the scattering profile, indicating the aggregation of ionic clusters in the densely functionalized AEM C-HBM-1.78. The image observed in TEM and AFM in combination with the SAXS profile demonstrated that the well-established ionic channels were formed within membrane C-HBM-1.78. This outstanding morphology is probably attributed to the specific structure of hyperbranched ionomer with densely distributed quaternary ammonium groups, which is believed to promote the self-assemble of quaternary ammonium groups to aggregate into ionic domains by narrowing the distance between quaternary ammonium groups during the membrane forming process. The formation of interconnected hydrophilic channels can thus be facilitated, which is beneficial for building effective ion conduction pathways, and guaranteeing high ion conductivity for membrane C-HBM-1.78. Despite that characteristic hydrophilic–hydrophobic phase separation is observed, it is apparent from the TEM and the SAXS data that no long-range, regular order of the ionic phase exists in membrane C-HBM-1.78 [35].

### 2.3. Ionic Conductivity and Water-Swelling Resistance Property

**Figure 5:**
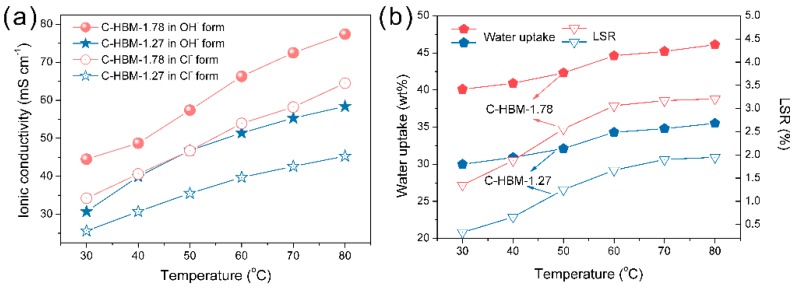
The ionic conductivity (**a**) and water uptake and linear swelling ratio (LSR) (**b**) as a function of temperature of membrane C-HBM-1.27 and C-HBM-1.78.

For a practical fuel cell application, ionic conductivity and water uptake is of particular importance. An ideal AEM should have high ionic conductivity and depressed water adsorption. The ionic conductivity for membrane C-HBM-1.27 and C-HBM-1.78 as a function of temperature is displayed in Figure 5a. Owing to the high sensitivity of hydroxide conductivity in the atmosphere, the chloride conductivity is measured and plotted as well. As expected, both the hydroxide conductivity and chloride conductivity increases with increasing temperature as a result of the enhanced dissociation efficiency and bigger water retention capacity. Higher ion content, that is, IEC value, indeed leads to higher conductivity. Specifically, the chloride conductivity for C-HBM-1.27 at 30 °C is 25.6 mS cm^−1^, while it rises to 39.7 mS cm^−1^ at 60 °C. For membrane C-HBM-1.78, higher chloride conductivity up to 34.2 mS cm^−1^ at 30 °C and 53.9 mS cm^−1^ at 60 °C is achieved.

**Figure 6:**
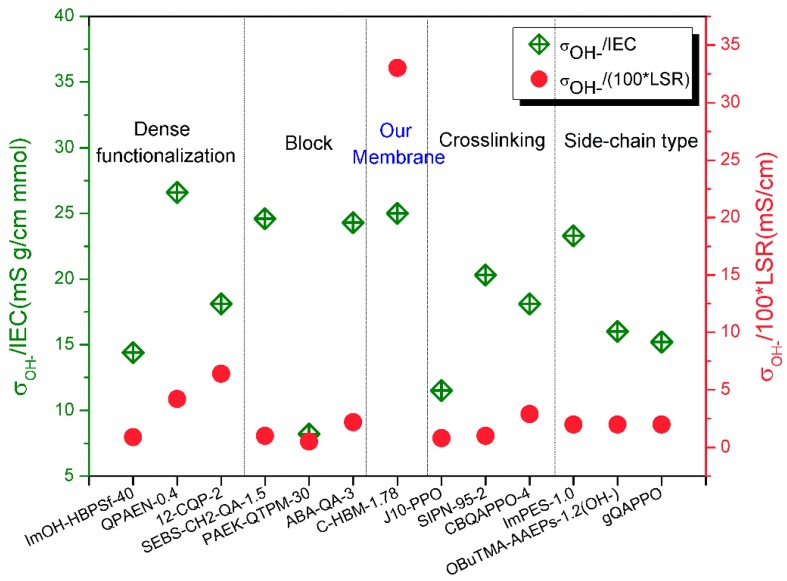
Comparison regarding effective hydroxide conductivity (σ_OH-_/IEC, σ_OH-_/LSR) of our membrane C-HBM-1.78 with reported densely functionalized, block, crosslinked, and side-chain-type membranes.

For better comparison, the hydroxide conductivity normalized on the basis of IEC (σ_OH–_/IEC) is put forward, which indicates the efficiency of quaternary ammonium groups in the membrane for transporting hydroxide ions. As shown in Figure 6, membrane C-HBM-1.78 displayed higher σ_OH-_/IEC of 25 than that of most published AEMs, including the side-chain-type [36,37,38], crosslinked [4,33,39], block [10,17,40], and densely functionalized AEMs [16,28,41], indicating its more efficient utilization of functional groups. This anion-conducting property should benefit from the well-developed microphase separated morphology along with interconnected hydrophilic channels, which can provide a smooth pathway for ion transportation. The high ionic conductivity is thus achieved, implying greater potential for applications in AFCs. However, as we all know, the high ion conductivity is strongly dependent on high water contents.

Introducing the cross-linking structure into AEMs is proven to be an effective method of restraining the water swelling. The water adsorption capacity and water swelling ratio are measured at the temperature range of 30–80 °C. As plotted in Figure 5b, both C-HBM-1.27 and C-HBM-1.78 showed increased water uptake with increasing temperature. Furthermore, higher water uptake is observed for membrane C-HBM-1.78 than that of C-HBM-1.27 owing to the higher ion content. For C-HBM-1.78, an acceptable water uptake of 46.1% and extremely low LSR of 3.2% at 80 °C was obtained. The excellent water-swelling resistance undoubtedly originated from the crosslinking structure and well established microphase separated morphology, which was confirmed by TEM, AFM, and SAXS. To highlight the outstanding advantages of the ionomer structure combining dense functionalization and crosslinking, the hydroxide conductivity and water-swelling resistance property at 30 °C of membrane C-HBM-1.78 are listed in Table 1 and compared with reported AEMs in Figure 6. Apparently, the σ_OH-_/water uptake for our membrane Cr-M-1.78 of 1.1 is much higher than most AEMs with the side-chain-type (0.5–0.7) [36,37,38], crosslinking (0.1–0.4) [4,33,39], and block (0.2–0.6) [10,17,40] architectures, indicating its higher utilization efficiency of water molecules. An outstanding σ_OH–_/LSR value of 33.0 for membrane C-HBM-1.78 is also obtained, which is around fifteen-fold higher than the side-chain-type (2.0) [36,37,38], crosslinking (0.8–2.9) [4,33,39], and block (0.5–2.2) [10,17,40] AEMs, and five-fold higher than the densely functionalized AEMs (0.9–6.4) [16,28,41]. In general, a great advantage of membrane C-HBM-1.78, including the higher conductivity in combination with largely depressed water swelling, is detected. This illustrates the effectiveness of combining dense functionalization and crosslinking to alleviate the trade-off effect between ionic conductivity and water swelling.

### 2.4. Alkaline Stability and Thermal Stability

**Figure 7:**
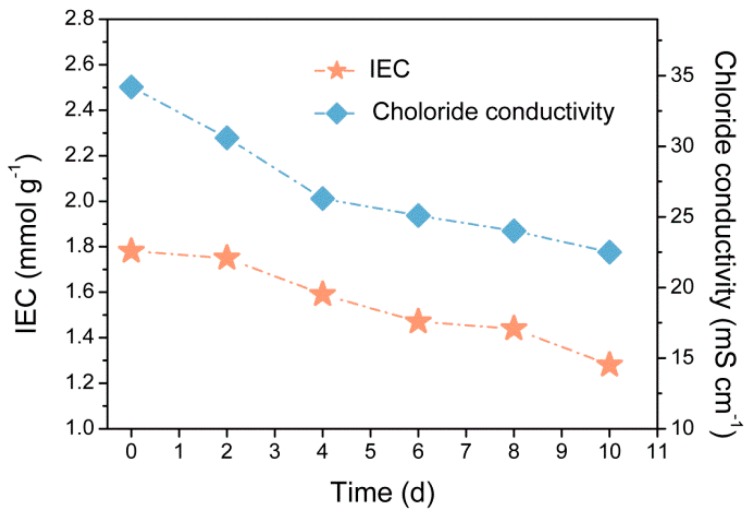
Time courses of chloride conductivity and IEC values of membrane C-HBM-1.78, in 1 mol L^−1^ NaOH aqueous solution at 60 °C. The membrane sample was converted into chloride form before conductivity measurement to avoid the influence of atmosphere CO_2_.

Fuel cells usually operate under harsh basic conditions and elevated temperatures, which requires robust alkali-resistance and good thermally stable AEM. The alkaline stability of membrane C-HBM-1.78 is determined by examining the variation of IEC values and chloride conductivity after immersing membrane samples in a 1 mol L^−1^ NaOH aqueous solution at 60 °C at different exposure times. The membrane samples maintained their toughness, flexibility, and appearance after stability tests as long as 240 h, suggesting no decomposition of aromatic main chains. Figure 7 shows the decline in IEC values and chloride conductivity of membrane C-HBM-1.78 with the conditioning time. The chloride conductivity of the treated membrane C-HBM-1.78 at the 10th day remains at 22.5 mS cm^−1^ at 30 °C, which satisfies the basic requirement of the hydroxide conductivity (over 10 mS cm^−1^) for fuel cell operation. The IEC values appeared as 1.28 mmol g^−1^, 72% of the retention after 10 days treatment was achieved. It has already been demonstrated that the quaternary ammonium groups tend to disintegrate in alkaline solution because of the displacement of the ammonium group by OH^−^ via a direct nucleophilic substitution and Hofmann elimination when β-H atoms are present. In the present study, there is no β-H atom; the C-HBM-1.78 was thus degraded mainly by the nucleophilic substitution in which the hydroxide ions attack the α-carbon of ammonium cations. The outstanding alkali-resistance performance should benefit from the well-established hydrophilic–hydrophobic separation and crosslinking structure, where OH^−^ is confined into hydrophilic domains and the aromatic rings can be well protected by the additional hydrophobic structure.

**Figure 8:**
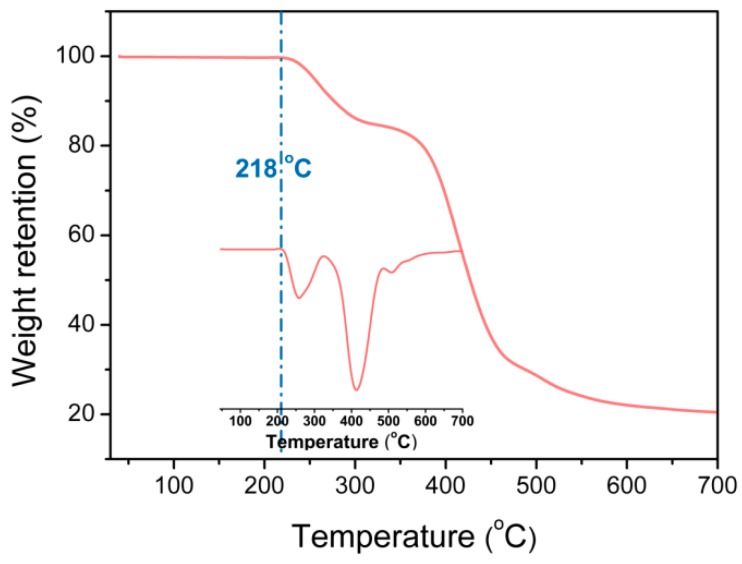
Thermogravimetic analysis (TGA) and differential thermal gravity (DTG, illustration) curves of membrane C-HBM-1.78.

The thermal stability of C-HBM-1.78 was investigated by thermogravimetic analysis (TGA) analysis. As shown in the TGA and differential thermal gravity (DTG) curve in Figure 8, a three-step degradation profile is observed for membrane C-HBM-1.78 from room temperature to 700 °C. The first weight loss occurred at around 218 °C, presumably corresponding to the degradation of the benzyltrimethylammonium chloride groups, and indicates the upper limit for practical application temperature of membrane C-HBM-1.78 in fuel cells. The weight loss between 218 and 700 °C is related to the degradation of the aromatic chains. In general, the thermal stability of C-HBM-1.78 can fulfill the practical application of fuel cells, which are typically operated at 60–80 °C.

## 3. Materials and Methods

### 3.1. Materials

The hyperbranched oligomer HB-PVBC was prepared and purified according to our previous procedure [34]. The chemical structure was examined by ^1^H NMR, and the molecular weight (Mn = 7560 g mol^−1^, Mw = 23629 g mol^−1^) and polydispersity (PDI = 3.13) were determined by GPC. The monomer 4-vinylbenzyl chloride, trimethylamine aqueous solution (30%), and N-methyl pyrrolidone (NMP) were purchased from Energy Chemical (Shanghai, China) and used as received. The initiator AIBN was purchased from Energy Chemical (Shanghai, China) and recrystallized from ethanol. Cuprous chloride (CuCl) was purchased from Energy Chemical (Shanghai, China) and purified according to the reported procedure [42]. Dimethyl amine aqueous solution (33 wt%), anhydrous magnesium sulfate (MgSO_4_), sodium chloride (NaCl), sodium sulfate (Na_2_SO_4_), and silver nitrate (AgNO_3_) were purchased from Sinopharm Chemical Reagent Co. Ltd. (Shanghai, China). Potassium chromate (K_2_CrO_4_) was purchased from Alfa Aesar (Shanghai, P.R. China) and potassium iodide (KI) was purchased from Aladdin (Shanghai, P.R. China). Unless otherwise noted, all reagents are of analytical grade and were used as received. Distilled water was used throughout the experiment.

### 3.2. Preparation of Monomer N,N-Dimethylbenzylamine (1) and Polyamine Poly(N,N-Dimethylbenzylamine) (2)

The monomer *N*,*N*-Dimethylbenzylamine was synthesized by treating 4-vinylbenzyl chloride with dimethyl amine at room temperature according to a modified procedure [43]. Specifically, to a dimethyl amine solution (33 wt%, 50 mL, 0.25 mol) in a 250 mL, three-necked, round-bottomed flask equipped with a magnetic stir bar, 4-vinylbenzyl chloride (25 mL, 0.18 mol) was added dropwise via a dropping funnel over a period of 3 h under vigorous stirring. The reaction was allowed to proceed at room temperature for another 3 h. Then, the organic layer was separated by a separating funnel and dried over anhydrous MgSO_4_, and the residue was vacuum distilled to obtain the monomer *N*,*N*-Dimethylbenzylamine from the mixture of *N*,*N*-Dimethylbenzylamine and poly(*N*,*N*-Dimethylbenzylamine) in 56% yield. ^1^H NMR was used to characterize the structure.

Poly(*N*,*N*-Dimethylbenzylamine) was prepared via radical polymerization using AIBN as an initiator and NMP as the solvent. In a typical polymerization procedure, *N*,*N*-Dimethylbenzylamine (1.36 g, 8 mmol), NMP (0.6 mL), and AIBN (0.013 g, 0.08 mmol) were introduced into a 25 mL, round-bottomed flask equipped with a magnetic stir bar. The reaction mixture was filled nitrogen for 20 min to remove oxygen prior to the polymerization process, and subsequently placed into an oil bath at 65 °C. After 12 h, the reaction was stopped and the mixture was diluted with NMP and precipitated into water. This crude product was further purified for two times, and finally vacuum dried at 50 °C for 24 h to give the white product poly(*N*,*N*-Dimethylbenzylamine). ^1^H NMR was utilized to prove its successful preparation and GPC was performed to characterize its molecular weight.

### 3.3. Quaternization of HB-PVBC (3) with Polyamine Poly(N,N-Dimethylbenzylamine) (2)

The densely functionalized AEM was fabricated via facile Menshutkin reaction of HB-PVBC and poly(*N*,*N*-Dimethylbenzylamine). Because of the particular structure of hyperbranched oligomer HB-PVBC with densely distributed benzylic chloride groups, the quaternization of HB-PVBC with polyamine poly(*N*,*N*-Dimethylbenzylamine) consequently yields a crosslinked AEM with densely arranged quaternary ammonium groups. In a typical procedure, to a solution of HB-PVBC (0.3 g) in NMP (6 mL), the solution of poly(*N*,*N*-Dimethylbenzylamine) (0.1 g) in NMP (2 mL) was slowly added under vigorous stirring. After stirring at room temperature for 10 min, the solution was directly cast on a flat, clean glass plate and then evaporated at 70 °C for 12 h, producing a transparent membrane. The membranes in OH^−^ form were obtained by immersing the membrane in NaOH aqueous solution (1 M) at room temperature for 24 h. The membrane sample was then washed thoroughly and immersed in distilled water for 48 h to remove residual NaOH before use.

### 3.4. Characterization

#### 3.4.1. Nuclear Magnetic Resonance (NMR) and Fourier Transform Infrared (FTIR)

The chemical structure of *N*,*N*-Dimethylbenzylamine, poly(*N*,*N*-Dimethylbenzylamine) and HB-PVBC was determined by ^1^H NMR (AVANCEII, 400MHz) with chloroform-d (CDCl_3_) as solvents. FTIR was performed on membrane sample and hyperbranched oligomer HB-PVBC on a TENSOR27 FT-IR Spectrometer (Germany) under ambient conditions with a resolution of 5 cm^−1^ and a wide spectral range of 4000–500 cm^−1^ to characterize its structure.

#### 3.4.2. Gel Permeation Chromatography (GPC)

The molecular weight and polydispersity (PDI = Mw/Mn) of poly(*N*,*N*-Dimethylbenzylamine) and HB-PVBC were determined by GPC on a PL 120 Plus (Agilent Technologies co., Ltd., China) equipped with a differential refractive index detector. The PL Gel Mixed Carbon 18 SEC columns connected in series were used to achieve the separation. Freshly prepared polymer samples in tetrahydrofuran (THF, HPLC grade) were passed through a 0.45 μm polytetrafluoroethylene syringe filter prior to injection. HPLC grade THF containing 0.03 wt% LiCl was used as the eluent at a flow rate of 1.0 mL min^−1^. The detecting system was calibrated with a 2 mg mL^−1^ polystyrene (Mw = 110K).

#### 3.4.3. Membrane Morphology Characterization

The microphase separated morphology of membrane C-HBM was examined by transmission electron microscopy (TEM), atomic force microscopy (AFM), and small angle X-ray scattering (SAXS). The membrane sample for TEM was prepared as follows: membranes were stained by soaking in a 1 mol L^−1^ potassium iodide (KI) aqueous solution at room temperature for 72 h, then washed with distilled water many times to remove the absorbed KI, and dried under vacuum at 40 °C. The stained membrane sample was then sectioned to yield slices with a thickness of 60–100 nm using a LEICA UC7FC7 ultramicrotome and coated on a copper grid. The electron micrograph was taken on a JEM-2100 transmission electron microscope operated at an accelerating voltage of 200 kV.

AFM observations in tapping mode were performed on a membrane sample in dry state with a veeco diInnova scanning probe microscope (SPM), using micro fabricated cantilevers with a force constant of approximately 20 N m^−1^.

SAXS measurement was carried out on the SAXSess mc2 X-ray scattering system (Anton Paar). SAXS measurement was performed with Cu Kα radiation operating at 2 kW (40 kV and 50 mA). The distance between the sample and detector was approximately 260 mm and the wavelength of X-rays was 1.542 Å. The exposure time was 30 min for the sample.

#### 3.4.4. Ion Exchange Capacity (IEC)

IEC was measured by Mohr’s method. The membrane sample was firstly soaked in NaCl aqueous solution (1 M) for 24 h, then washed with distilled water to remove the absorbed NaCl and dried to a constant weight and weighed as W_dry_. Finally, the membrane was immersed in Na_2_SO_4_ aqueous solution (0.5 M) for 24 h to exchange Cl^−^ from the membrane with SO_4_^2−^. The released Cl^−^ ions were then titrated with AgNO_3_ aqueous solution (0.05 M) using K_2_CrO_4_ as a colorimetric indicator. The IEC value can thus be calculated from the amount of AgNO_3_ consumed in the titration process and the mass of the dry membrane in Cl^−^ form, as shown in Equation (1).

(1) $$\mathrm{IEC}\left(mmol/g\right)=V_{AgNO_{3}}\times C_{AgNO_{3}}/W_{dry}$$

#### 3.4.5. Hydroxide Conductivity and Chloride Conductivity

The OH^−^ conductivity and chloride conductivity of membrane C-HBM were measured using a four-point probe technique on an Autolab PGSTAT 302N (Eco Chemie, Switzerland) equipped with a Teflon cell. During the measurement, the membrane sample was set into the Teflon cell, in which it was in contact with two current-collecting electrodes and two potential-sensing electrodes. Then, the cell was completely immersed in distilled water, and the impedance spectrum in galvanostatic mode and with an ac current amplitude of 0.1 mA over frequencies ranging from 1 MHz to 100 Hz was collected. Bode plots were used to determine the frequency region over which the magnitude of the impedance was constant. The ionic resistance of membrane was then obtained from a Nyquist plot and the ionic conductivity was calculated according to Equation (2):

(2) σ=L/RWd

where L is the distance between two potential-sensing electrodes (here, 1 cm); R is the absolute ohmic resistance of the membrane sample; and W (here, 1 cm) and d are the width and thickness of the membrane, respectively.

#### 3.4.6. Water Uptake and Linear Swelling Ratio (LSR)

Membrane samples in Cl^−^ form were immersed in distilled water at given temperatures for 24 h, then removed, and the membrane surfaces were quickly wiped dry with tissue paper. Water uptake and LSR of the membranes were calculated from the mass and length of wet and dry samples as follows:

(3) $$WU\left(wt\%\right)=\left(W_{wet}-W_{dry}\right)/W_{dry}\times 100\%$$

(4) $$LSR\left(\%\right)=\left(L_{wet}-L_{dry}\right)/L_{dry}\times 100\%$$

where *W_wet_* and *L_wet_* are the weight and length of the wet membrane, and *W_dry_* and *L_dry_* are those of the dry membrane after dried at 60 °C in a vacuum oven for 24 H, respectively.

#### 3.4.7. Dynamic Mechanical Analyzer

The mechanical property of C-HBM at fully hydrated state was tested on a Q800 dynamic mechanical analyzer (TA Instruments) at a stretch rate of 0.5 N min^−1^.

#### 3.4.8. Thermogravimetic Analysis (TGA)

The thermal behavior of the membrane C-HBM was examined on a Perkin-Elmer Pyris-1 analyzer (USA) from 30 to 700 °C at a heating rate of 10 °C min^−1^ under a nitrogen atmosphere.

#### 3.4.9. Alkaline Stability

To assess the alkali-resistance property of C-HBM, membrane samples were immersed in 1 mol L^−1^ NaOH aqueous solutions at 60 °C for 10 days. They were then taken out and thoroughly washed with distilled water prior to the measurement of IEC values and ionic conductivity. The chloride conductivity was measured by exchanging OH^−^ with Cl^−^ thoroughly to avoid the influence of atmosphere CO_2_.

## 4. Conclusions

In conclusion, to alleviate the trade-off effect between ionic conductivity and water swelling, a crosslinking hyperbranched AEM was designed and fabricated, aiming to combine both advantages of dense functionalization and crosslinking. As expected, a much higher ratio of hydroxide conductivity to water swelling than that of the common side-chain-type, block, and single dense functionalization and single crosslinking AEMs was observed. Additionally, outstanding alkaline stability and thermal stability were also obtained for membrane C-HBM-1.78. The combination of excellent hydroxide conductivity and outstanding water-swelling resistance makes membrane C-HBM-1.78 attractive as AEM materials for fuel cell applications. Furthermore, this strategy proposed herein opens up new possibilities for overcoming the trade-off effect between ionic conductivity and water swelling for ion exchange membranes.

## Figures and Tables

**Table 1:** Comparison regarding ion exchange capacity (IEC), hydroxide conductivity, water uptake, and linear swelling ratio (LSR) of C-HBM-1.78 with reported membrane samples.

Membrane Sample	Structure	Titrated IEC (mmol g^−1^)	σ_OH__–_	Water Uptake (wt%)	LSR (%)	σ_OH__–_/IEC	σ_OH–_/(100*Water uptake)	σ_OH__–_/(100*LSR)	Reference
C-HBM-1.78 ^a^	Crosslinking, Dense functionalization	1.78	44.5	40.1	1.4	25.0	1.1	33.0	This work
ImPES-1.0 ^a^	Side chain type	1.83	42.7	83.9	21.7	23.3	0.5	2.0	[36]
OBuTMA-AAEPs-1.2(OH^−^) ^b^		1.87	30.0	62.0	14.7	16.0	0.5	2.0	[37]
gQAPPO ^a^		1.78	27.0	40.0	13.2	15.2	0.7	2.0	[38]
CBQAPPO-4 ^b^	Crosslinking	2.37	43.0	110.0	15.0	18.1	0.4	2.9	[33]
J10-PPO *^c^*		2.08	24.0	228.0	32.0	11.5	0.1	0.8	[4]
SIPN-95-2 ^a^		1.75	35.5	213.1	37.5	20.3	0.2	1.0	[39]
ABA-QA-3 ^a^	Block	1.81	44.0	86.3	20.0	24.3	0.5	2.2	[10]
SEBS-CH_2_-QA-1.5 ^a^		1.23	30.2	47.5	29.6	24.6	0.6	1.0	[17]
PAEK-QTPM-30 ^c^		1.58	13.0	88.6	27.5	8.2	0.2	0.5	[40]
12-CQP-2 ^b^	Dense functionalization	1.40	25.4	10.7	4.0	18.1	2.4	6.4	[16]
ImOH-HBPSf-40 ^a^		2.16	31.0	143.9	36.3	14.4	0.2	0.9	[41]
QPAEN-0.4 ^a^		1.78	47.3	34.2	11.2	26.6	1.4	4.2	[28]

^a^ The σ_OH-_, water uptake, and LSR were tested at 30 °C; ^b^ the σ_OH-_, water uptake, and LSR were tested at 25 °C; ^c^ the σ_OH-_, water uptake, and LSR were tested at 20 °C.

## Abbreviations

PEMs	Proton exchange membranes
AEMs	Anion exchange membranes
AFCs	Alkaline fuel cells
PEMFCs	Proton exchange membrane fuel cells
NMP	N-methyl pyrrolidone
HB-PVBC	Hyperbranched poly(4-vinylbenzyl chloride)
AIBN	2,2’-azodiisobutyronitrile
ATRP	Atom transfer radical polymerization
IEC	Ion exchange capacity
LSR	Linear swelling ratio
GPC	Gel permeation chromatography
NMR	Nuclear magnetic resonance
FTIR	Fourier transform infrared
TEM	Transmission electron microscopy
AFM	Atomic force microscopy
SAXS	Small angle X-ray scattering
DSC	Differential scanning calorimetry
TGA	Thermogravimetic analysis
